# Challenges and Solutions in Deploying Systematized Nomenclature of Medicine—Clinical Terms in the Chinese Healthcare Context

**DOI:** 10.1002/hcs2.70069

**Published:** 2026-04-21

**Authors:** Ge Wu, Jiale Nan, Yanmei Chen, Chao Liu, Taotao Fu, Xudong Lu, Yani Chen, Zhirong Zeng, You Wu, Mengchun Gong

**Affiliations:** ^1^ School of Biomedical Engineering Guangdong Medical University Zhanjiang China; ^2^ College of Biomedical Engineering & Instrumentation Zhejiang University Hangzhou China; ^3^ School of Artificial Intelligence Dalian Maritime University Dalian China; ^4^ GMC Lab, School of Biomedical Engineering Guangdong Medical University Dongguan China; ^5^ Institute for Hospital Management, Tsinghua Medicine Tsinghua University Beijing China; ^6^ Global Health Institute Xi'an Jiaotong University Xi'an China

**Keywords:** data standardization, generative AI, healthcare big data, healthcare IT, large language models, SNOMED CT

## Abstract

Systematized nomenclature of medicine—clinical terms (SNOMED CT), one of the most comprehensive clinical terminology systems, is pivotal in enhancing healthcare interoperability, clinical data governance, and medical artificial intelligence (AI) development globally. In China, with the rapid growth of large‐scale models and an increasing emphasis on transforming the intrinsic value of healthcare data, the absence of a nationally unified clinical terminology standard poses significant challenges. This commentary provides an in‐depth analysis of the benefits of SNOMED CT for global healthcare, examines the critical deficiencies in Chinese healthcare big data and AI development due to the lack of standardized terminology, and outlines the technical, administrative, and educational challenges encountered in deploying SNOMED CT within Chinese environments. Special emphasis is laid on the potential of advanced large language models in facilitating the mapping of Chinese clinical data to SNOMED CT. We further discuss the necessity of high‐quality data standardization in advancing medical AI in China. Finally, key conclusions and a roadmap for overcoming these challenges are proposed.

## Graphical abstract

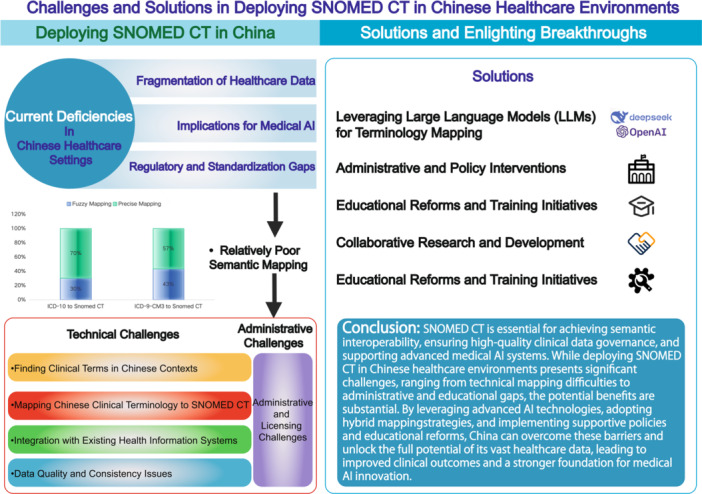

**Abbreviation**
SNOMED CT: systematized nomenclature of medicine – clinical terms

## 1 Introduction

The contemporary healthcare landscape is increasingly shaped by digital transformation, the exponential growth of health data, and the revolutionary application of large models, such as DeepSeek. We need a comprehensive clinical terminology to serve as the central hub for effective data exchange, clinical decision support, and advanced analytics in healthcare, ensuring semantic interoperability across systems. Systematized Nomenclature of Medicine—Clinical Terms (SNOMED CT) has emerged as a cornerstone of clinical ontologies, facilitating not only consistent data recording and retrieval but also supporting research, public health reporting, and the implementation of sophisticated artificial intelligence (AI) algorithms [1, 2].

In China, the healthcare system is undergoing rapid evolution with significant investments in big data initiatives and AI‐driven innovations, especially with the fast implementation of DeepSeek and related AI applications. Despite these advances, a critical barrier remains—the lack of a nationally unified clinical terminology standard [3]. This gap severely impedes the effective integration and utilization of vast amounts of heterogeneous clinical data, ultimately limiting the potential of AI applications in healthcare [4]. Moreover, technical challenges and administrative constraints, such as the absence of an official SNOMED CT membership and restrictive licensing issues, complicate the deployment of this international standard in the Chinese healthcare environment. However, some use cases were established under affiliate licenses.

Recent work by leading Chinese experts has underscored the urgency of addressing these challenges. These studies not only illustrate innovative approaches (such as attention‐based semantic matching for automatic SNOMED CT coding) [5] but also demonstrate the feasibility of cloud‐based systems for integrating and standardizing diverse clinical data [6].

## 2 The Global Significance of SNOMED CT

The increasing digitalization of healthcare requires structured and standardized medical data to enhance interoperability and support AI‐driven innovations [7]. SNOMED CT provides a hierarchical, semantically rich framework for encoding clinical information, enabling efficient data exchange across systems, improving patient care, and facilitating large‐scale research initiatives [8]. Many countries, including the United States, many European Union member countries, the United Kingdom, and some Asian and Oceanian countries such as Republic of Korea, Singapore, India, and Australia, have successfully integrated SNOMED CT into their national healthcare systems, ensuring interoperability between electronic health records (EHRs) and research databases, and enabling multi‐national real‐world evidence studies with the sample sizes over millions [9]. Among these countries, the United Kingdom has reported improvements in data quality and user satisfaction through SNOMED CT, whereas the United States has focused on automated coding and cross‐institutional data sharing [10]. Besides, they have shared their experience mapping EHRs to SNOMED CT and reported various challenges. In practice, Western countries were more concerned with technical challenges such as interoperability and contextual modeling, while Asian countries focused on expanding localization and ensuring terminology compatibility. Data quality, version synchronization, and privacy compliance were common issues across regions.

### 2.1 Interoperability and Clinical Data Governance

Interoperability is a fundamental requirement for the seamless exchange of clinical information across different healthcare systems. SNOMED CT provides a unified language that reduces ambiguities in clinical documentation, thereby improving patient safety, enabling further data analytics, and supporting high‐quality AI solution development and application. Standardization of terminology is critical for clinical data governance, ensuring that data collected from disparate sources maintains consistency and reliability over time [11]. Moreover, the use of SNOMED CT enhances data quality, which is indispensable for secondary uses such as epidemiological research and quality assurance [8].

### 2.2 Facilitating the Development of Medical AI

The development of AI in healthcare relies heavily on high‐quality, well‐structured data. SNOMED CT's comprehensive ontology enables machine learning algorithms to process and interpret clinical data effectively. Numerous studies in different language environments have demonstrated that standardized terminologies not only streamline data integration but also enhance the performance of AI models by providing a common semantic framework [7]. As healthcare systems worldwide move toward data‐driven decision‐making, integrating SNOMED CT into EHRs and health information systems is a critical step toward realizing the full potential of generative AI technologies, especially for large language models [12].

## 3 Real‐World Conditions of the Real‐World Data in China

China's healthcare system has witnessed rapid growth in the volume of clinical data and institutional deployment of DeepSeek. However, this growth has not been matched by the establishment of a cohesive framework for data standardization, resulting in fragmented and heterogeneous real‐world datasets [13].

### 3.1 Fragmentation of Healthcare Data

Chinese healthcare data is often collected from a pool of disconnected sources, including public hospitals, private clinics, community health centers, and individuals. Without a unified standard, data from these diverse sources is recorded using varying terminologies and formats, leading to significant issues in data integration and interoperability. The absence of a common clinical language weakens the efficiency of compiling, analyzing, and deriving meaningful insights from these datasets [14].

### 3.2 Implications for Medical AI

Medical AI depends on large, high‐quality datasets for training and validation. The heterogeneity of Chinese clinical data increases the noise within datasets and limits the generalizability of AI models. Studies have indicated that inconsistent terminologies and data formats decrease predictive accuracy and increase bias in AI applications [15]. Thus, despite the widespread installation of DeepSeek inside Chinese hospitals, the lack of standardized clinical terminologies such as SNOMED CT contributes to a slower pace of innovation and adoption of AI technologies in serious clinical scenarios in Chinese healthcare settings.

### 3.3 Regulatory and Standardization Gaps

While the Chinese government is actively promoting policies to leverage healthcare data as a novel production factor, these efforts are undermined by the absence of nationwide clinical terminology standards. The lack of a unified ontology affects data interoperability and impedes the regulatory framework needed to ensure data quality and patient safety [16]. Recent policy reviews have underscored the need to establish unified standards as a foundational step toward an ecosystem of using high‐quality clinical datasets as a novel production factor to drive novel production power and boost the economy.

## 4 Challenges in Deploying SNOMED CT in China

Despite the global recognition of SNOMED CT's value, its implementation in China encounters substantial obstacles, which can be categorized into technical difficulties and administrative limitations.

### 4.1 Difficulty in Gathering Clinical Terms in Chinese Contexts

**Figure 1:**
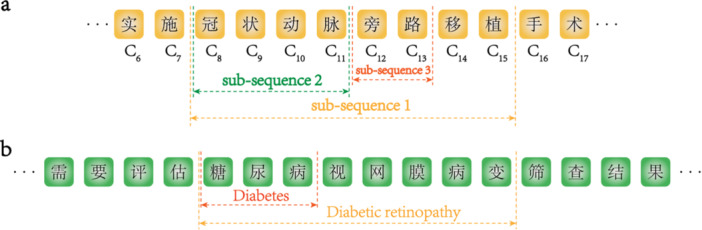
Sophisticated linguistic problems in Chinese contexts. (a) The example indicates that the ground‐truth entity (entitled “subsequence 1”) may be mistakenly cut into separate entities owing to different semantics. (b) The example shows the phenomenon of ambiguity, in which diabetes is legal in both segmented dependency relations, but only diabetic retinopathy is the ground‐truth entity under this Chinese context.

Yu et al. [17] conducted a study on 8362 medical terms from the clinical terminology library of the National Health Service system, revealing that 7925 terms could be successfully mapped to SNOMED‐CT, achieving a coverage rate of 94.77%. In contrast, Zhang et al. [15] developed a system for extracting medical terms from clinical texts, identifying 59,461 Chinese clinical terms, of which only 3729 (6.27%) had exact matches in the Chinese versions of SNOMED‐CT and ICD‐10. In addition to data entry standards, the significant disparity in matching rates between these studies underscores the critical challenge of identifying relevant terms in Chinese medical records. This issue can only be solved by decoding the path of semantic generation in Chinese contexts. Distinguishing different strategies that Chinese and English texts use to generate semantic words and sentences will largely promote our understanding of the language expressiveness in Chinese health records and synonym merging of SNOMED CT Chinese extension. In Chinese contexts, the smallest semantic unit “character” (which may be meaningful by itself) generates meaningful words and sentences to describe phenomena and findings in clinical settings. The ambiguity problem (Figure 1), caused by incorrect character segmentation [16], requires advanced strategies and algorithms to accurately determine the right expression of medical acts.

### 4.2 Mapping Chinese Clinical Terminology to SNOMED CT

**Figure 2:**
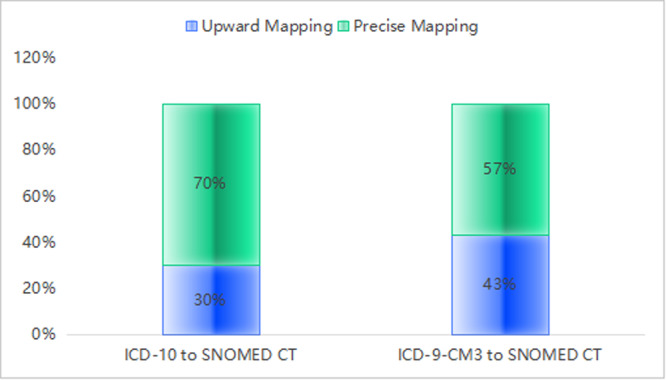
Semantic mismatching of SNOMED CT terminology set with the Chinese version of ICD‐10 and ICD‐9‐CM3.

One of the primary technical challenges lies in the inherent difficulty of mapping Chinese clinical terminologies to the SNOMED CT framework. Linguistic and cultural differences mean that many concepts in Chinese clinical practice lack direct counterparts in SNOMED CT. The ICD‐10 Chinese terminology set contains 37,109 standardized terms, while the ICD‐9‐CM3 Chinese terminology set includes 13,654 terms. A mapping analysis conducted by our team quantified the semantic matching proportion between SNOMED CT and two commonly used terminology systems in China, containing 368,244 standardized real‐world clinical terms (131,634 original diagnosis terms, 54,190 original procedure terms, and 182,420 original examination terms) collected from 18 hospitals in China. The study revealed that 30% of terms in the Chinese version of ICD‐10 and 43% in ICD‐9‐CM3 were too fuzzy to match proper counterparts or did not exist in SNOMED CT (Figure 2). Developing accurate and comprehensive mappings requires linguistic translation and a deep understanding of clinical context [7]. Recent studies in China have begun addressing this issue. For example, Lu Xudong and colleagues have developed an attention‐based semantic matching algorithm that automatically codes Chinese clinical terms with SNOMED CT codes (a total of 29,960 manually coded Chinese clinical terms, 30,040 unmatched Chinese clinical terms), achieving state‐of‐the‐art performance in terms of accuracy (0.905) and precision (0.856), with a recall of 0.518, and an *F*‐measure of 0.645 [5]. Additionally, pilot studies on the translation and localization of SNOMED CT have demonstrated promising results, indicating that with careful adaptation, the international standard can be effectively tailored for local clinical practice [12].

### 4.3 Integration With Existing Health Information Systems

Integrating SNOMED CT with legacy health information systems remains another significant hurdle. Many existing EHR systems in China were developed without consideration for international terminologies. Upgrading these systems to support SNOMED CT requires substantial modifications, including database restructuring, software updates, vocabulary management, and personnel retraining [8]. This complexity is a deterrent for many institutions, particularly those with limited IT resources.

### 4.4 Data Quality and Consistency Issues

Even with effective mappings, ensuring the consistent application of SNOMED CT across diverse clinical settings is challenging. Variations in data entry practices, incomplete documentation, inconsistent term usage, semantic ambiguity, low annotation quality of clinical data, and data redundancy, as well as conflicts with contextual representation, can undermine the benefits of semantic interoperability. The risk associated with changing the existing data report workflows for hospital management and insurance reimbursement also deters the IT management department into deploying a fundamental vocabulary service engine. Without high‐quality data input, the potential improvements in clinical decision support and AI‐driven analytics may remain unrealized, even with the novel capability in the processing of unstructured data by DeepSeek [7].

### 4.5 Applying Large Language Modeling (LLM) for Mapping Unstructured Text to Codes

Mapping unstructured clinical texts, such as medical records and imaging reports, to SNOMED CT codes through LLM is an important technical path to improve medical data standardization. However, it also faces many challenges. In the technical aspect, the semantic complexity of medical texts (e.g., diverse unstructured expressions, long‐term descriptions in complex cases, and multilevel relationships of SNOMED CT coding) means that the model needs to accurately capture the context and is required to have the ability to reason. Moreover, the training data coverage is insufficient. The medical corpus of general LLMs, such as DeepSeek, has a low proportion and weak generalization ability for rare diseases and specialized terms. Lacking high‐quality annotated data for training is one of the key challenges. During practical application, compliance and privacy risks should also be considered. Model training must comply with the local law for data and privacy protection, and further study is required to analyze and quantify the privacy risks of the application of LLM mapping.

### 4.6 Administrative and Licensing Challenges

**Figure 3:**
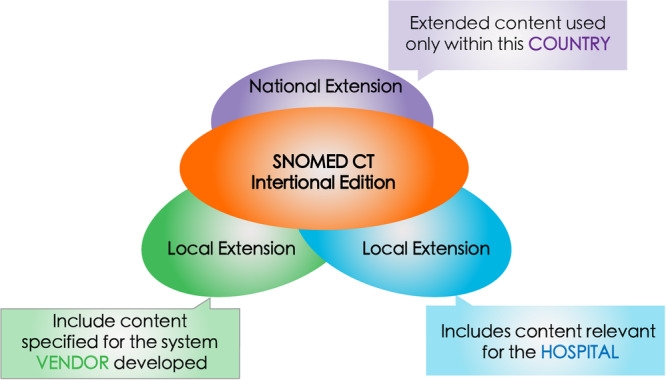
National extension content is used only within the country and may vary in different countries. Local extension includes content for system vendors, as well as hospitals are included in their national extension. The lack of issued affiliated licenses at the national level restricts collaborative extension for Chinese healthcare practices.

A critical administrative barrier is China's lack of formal membership in SNOMED CT. Membership in SNOMED International (Figure 3) provides a country with the right to use, disseminate, and modify SNOMED CT. However, China's nonmembership status limits access to localized versions of SNOMED CT and restricts collaborativse adaptation efforts for Chinese healthcare practices. Current licensing frameworks for SNOMED CT do not readily accommodate China's unique regulatory and commercial environment, and the costs associated with licensing, combined with usage restrictions in both public and private sectors, further complicate SNOMED CT deployment [8]. Many institutions have acquired their SNOMED CT affiliate licenses through third‐party organizations, such as companies, but they lack the authority to translate SNOMED CT or submit concept development inquiries directly to SNOMED International.

Moreover, effective integration of SNOMED CT also requires alignment with national policies and regulatory frameworks. Without clear governmental policies, healthcare institutions may be reluctant to invest in the necessary infrastructure. Technical innovation investment by healthcare IT companies may also be insufficiently validated since there is no clear return prediction. Policy reform addressing technical and administrative dimensions is essential for successful implementation.

### 4.7 Educational Gaps in Chinese Medical Informatics

A less often discussed yet equally critical challenge is the inadequacy of medical informatics education in China, particularly regarding clinical terminologies and implementing such standards in healthcare IT systems. Traditional medical informatics education in China has predominantly focused on IT system development and management, literacy and archiving, and basic coding skills. However, there has been relatively insufficient emphasis on the theoretical foundations of terminology systems. Courses on ontology, semantics, and data standardization are often absent from many medical curricula [3]. Consequently, future informaticians and practicing clinicians are frequently ill‐equipped to effectively utilize complex systems like SNOMED CT.

Deficiencies in medical informatics education have far‐reaching consequences. Clinicians may struggle to adopt new technologies, especially when there is a huge institutional effort to deploy novel technologies such as DeepSeek, and researchers may find it challenging to leverage large, heterogeneous datasets for meaningful analysis. Without robust educational programs and continuous professional development, the transformative potential of SNOMED CT—and by extension, standardized data for AI—will remain underexploited in the Chinese healthcare context.

The rapid pace of technological innovation in healthcare demands a workforce proficient in both clinical practice and data science. In contrast to the Clinical Informatics Fellowship training system in the United States, current training programs in Chinese medical institutions seldom offer interdisciplinary courses that bridge clinical medicine and informatics. This gap is particularly significant in data standardization and vocabulary control, where a combined understanding of medical and computational knowledge is required to facilitate accurate data mapping and interpretation.

## 5 Potential Solutions

Addressing the multifaceted challenges in deploying SNOMED CT in China requires a coordinated approach that integrates technical innovation, administrative reform, and educational advancement. The emergence of large language models, with localized DeepSeek‐R1 as the prominent solution, offers a particularly promising avenue.

### 5.1 Use Cases of SNOMED CT Implementation in China and Lessons Learned

Complementing the technical and administrative challenges, recent Chinese research offers valuable insights into addressing these issues. For instance, research has demonstrated the feasibility of using cloud‐based systems to integrate heterogeneous clinical data during the COVID‐19 pandemic [6], enabling multinational real‐world evidence studies that cross language barriers [9, 18] and generating high‐quality decision‐supporting tools [19]. The ICD terminology set mainly involves diagnostic‐related terms, while SNOMED CT contains a wide range of domain axial information, providing more precise descriptions of the novel disease, such as medications, symptoms and their severity. This helped the CDC better understand the disease development and epidemic control. The underlying principles of data integration, standardization, and interoperability directly apply to SNOMED CT deployment. Such work emphasizes that robust digital infrastructures and agile regulatory frameworks can accelerate the standardization process in China.

Standardized data minimizes ambiguities and inconsistencies that arise from heterogeneous data sources. Accurate, consistent data are essential for training and deploying robust AI models capable of reliable clinical decision support. Implementing SNOMED CT across healthcare institutions in China can significantly enhance the quality of health data repositories [20]. Although China lacks formal membership in SNOMED CT, it can promote SNOMED CT in hospital information construction by granting affiliate licenses through qualified enterprises. A unified clinical terminology enables seamless data integration from multiple sources, facilitating large‐scale research studies that span various institutions inside the country. Such interoperability supports collaborative research and accelerates medical discovery [21]. The transformative potential of medical AI is closely linked to data quality. High‐quality, standardized datasets allow AI systems to achieve superior accuracy and reliability, leading to enhanced diagnostic support, personalized treatment recommendations, and improved patient outcomes. Investing in data standards and standardization is not merely a technical necessity but a strategic imperative for advancing healthcare innovation in China.

### 5.2 Leveraging Large Language Models for Terminology Mapping

Advances in natural language processing and large language models (e.g., GPT‐series, BERT‐based models, DeepSeek) have shown significant potential in bridging linguistic and terminological gaps [22]. These models can be trained on extensive Chinese clinical corpora to develop semantic representations that facilitate the mapping of local terminologies to SNOMED CT concepts [7, 11]. By automating parts of the mapping process, LLMs can reduce manual labor and improve both the accuracy and consistency of terminology conversion. However, expert oversight remains indispensable. A hybrid strategy—combining AI‐driven mapping with domain expert validation—can enhance the quality of mappings while accelerating the overall process [13]. Such an approach ensures that ambiguous cases are flagged and resolved by clinicians and informaticians, preserving semantic accuracy.

### 5.3 Administrative and Policy Interventions

Overcoming licensing and membership challenges will require active engagement between Chinese healthcare authorities and SNOMED International. Members of SNOMED CT hold the national license for SNOMED CT, which makes the clinical terminology free for use in their respective countries. More than 20 hospitals in China are using SNOMED CT under the affiliate licenses. Strategic partnerships and favorable licensing negotiations are critical steps toward broader adoption, and national licensing ensures that members have full access to the product set and that the SNOMED CT encoded clinical information is available to all relevant stakeholders. According to international experiences, other policies should be set up to facilitate the nationwide implementation of SNOMED CT, including funding for IT infrastructure upgrades, training initiatives, and research projects centered on data standardization in Chinese hospitals.

### 5.4 Educational Reforms and Training Initiatives

Chinese medical schools and informatics programs must revise their curricula to include comprehensive courses on clinical terminologies and ontologies to address educational gaps. Interdisciplinary programs that integrate clinical medicine, computer science, and data analytics will cultivate a new generation of experts capable of managing the complexities of data standardization. Targeted training for current clinicians and health information managers is also necessary to facilitate the transition to standardized practices. Although China has not publicly set up a “SNOMED CT team,” a substantive guidance formulation and implementation mechanism has been formed through the existing medical informatization management framework and pilot project collaboration. In addition, national projects such as the “National Key Research and Development Program of China” provide funding for information construction, and these funds also support training and education related to SNOMED CT.

### 5.5 Collaborative Research and Development

Encouraging collaborative research among academic institutions, healthcare providers, and technology companies will foster innovation in developing mapping tools, integration platforms, and training programs, benefiting clinical laboratory and scientific research. International collaborations can bring diverse expertise and help tailor global standards such as SNOMED CT to the unique needs of the Chinese healthcare system [23, 24]. Such research efforts address immediate technical and administrative challenges and contribute to the long‐term evolution of medical informatics in China.

## 6 Conclusions

SNOMED CT is essential for achieving semantic interoperability, ensuring high‐quality clinical data governance, and supporting advanced medical AI systems. Its comprehensive structure enhances clinical documentation and improves data usability for research and clinical decision support. Deploying SNOMED CT in Chinese healthcare environments presents significant challenges, ranging from technical difficulties to administrative and educational gaps, and the potential benefits are substantial. By leveraging advanced AI technologies, adopting hybrid mapping strategies, and implementing supportive policies and educational reforms, China can overcome these barriers and unlock the full potential of its vast healthcare data, leading to improved clinical outcomes and a stronger foundation for medical AI innovation.

## Author Contributions

**Ge Wu:** writing – original draft (equal), writing – review and editing (equal). **Jiale Nan:** writing – original draft (equal), writing – review and editing (equal). **Yanmei Chen:** data curation (equal), methodology (equal). **Chao Liu:** project administration (equal), supervision (equal). **Taotao Fu:** formal analysis (equal), visualization (equal). **Xudong Lu:** validation (equal). **Yani Chen:** methodology (equal). **Zhirong Zeng:** resources (equal), supervision (equal). **You Wu:** validation (equal). **Mengchun Gong:** conceptualization (equal), funding acquisition (equal), supervision (equal).

## Ethics Statement

The authors have nothing to report.

## Consent

The authors have nothing to report.

## Conflicts of Interest

The authors declare no conflicts of interest.

## Data Availability

Data sharing not applicable to this article as no datasets were generated or analyzed during the current study.
